# The Metabolic Calibration of Female Immune Plasticity: From X-Linked Vulnerability to Precision Metabotyping

**DOI:** 10.3390/biology15070558

**Published:** 2026-03-31

**Authors:** Zhengsha Huang, Jianwei Ren, Qipeng Shu, Yuntao Tang, Jia Zhang, Weizhe Yu, Chenxi Zhang, Yafang Pang, Lu Liu, Jiayue Han, Youfan Zhang, Weizhou Wang, Shangze Li

**Affiliations:** 1 College of Medicine, Xizang University, Lhasa 850000, China; 18886207417@163.com (Z.H.); rjw@utibet.edu.cn (J.R.); liulu1648@163.com (L.L.); 2 College of Medicine, Chongqing University, Chongqing 400030, China; qipengshu@cqu.edu.cn (Q.S.); 13397221357@163.com (Y.T.); zhangjia011223@163.com (J.Z.); 202437021011@stu.cqu.edu.cn (C.Z.); 202337131095@stu.cqu.edu.cn (J.H.); 202337131080@stu.cqu.edu.cn (Y.Z.); weizhouwang27@163.com (W.W.)

**Keywords:** sexual dimorphism, xist ribonucleoprotein, regulatory T cells, histone lactylation, microbiota-derived metabolites, metabotyping, immune homeostasis

## Abstract

The female immune system operates within an evolutionary stability-plasticity trade-off, where the physiological demands of pregnancy necessitate a considerable degree of regulatory T cell (Treg) plasticity. This essential flexibility, however, inherently lowers the threshold for autoimmune dysregulation. Recent mechanistic evidence has identified Xist ribonucleoprotein complexes as female-specific autoantigens that constitutively sensitize innate sensors, thereby establishing an intrinsic state of autoimmune priming. This review introduces the Metabolic Calibration Hypothesis, proposing that commensal microbiota-derived metabolites function as essential extrinsic stabilizers specifically required to maintain female immune homeostasis. Beyond canonical short-chain fatty acid signaling, we synthesize emerging evidence regarding host-microbiota metabolic integration, emphasizing the roles of histone lactylation in the reproductive tract and the ligand-specific activation of nuclear receptors by secondary bile acids in orchestrating Treg fate. We posit that female immune pathologies, ranging from systemic autoimmunity to gynecologic malignancies, represent a context-dependent dysregulation or co-option of this metabolic calibration. Ultimately, we discuss the clinical necessity of transitioning from generic biotic interventions toward Sexual Dimorphic Metabotyping, providing a precision framework to restore physiological tolerance and manage sex-biased immune pathologies.

## Simple Summary

Biological females generally exhibit more robust immune responses than males, which provides superior protection against infections but significantly increases the risk of autoimmune diseases such as lupus. This review explores why this disparity exists by introducing the Metabolic Calibration Hypothesis. We argue that the female immune system is naturally poised for action due to factors on the X chromosome, and it requires specific chemical signals from gut and vaginal bacteria to remain stable and avoid attacking the host. We detail how these microbial signals, particularly butyrate and lactate, act as essential regulators of immune cell function. By examining how this balance changes during pregnancy, autoimmunity, and cancer, we highlight the limitations of current one-size-fits-all treatments. We propose a new precision medicine approach, termed Sexual Dimorphic Metabotyping, to develop therapies tailored to the unique biological and metabolic needs of the female immune system.

## 1. Introduction

The human immune system exhibits a profound and conserved sexual dimorphism, a biological reality that has historically been underexplored in clinical immunology but is now recognized as a primary determinant of health outcomes [1,2,3,4,5]. Epidemiological data consistently demonstrate that biological females mount significantly more robust innate and adaptive immune responses to a broad spectrum of pathogens compared to males. This heightened reactivity translates into lower viral loads during infections such as SARS-CoV-2 and HIV, as well as superior efficacy of vaccines against influenza and hepatitis B [6,7]. However, this immunological vigor is associated with a trade-off: a marked susceptibility to autoimmune diseases. The sexual disparity is most striking in Systemic Lupus Erythematosus (SLE), which presents with a female-to-male ratio of approximately 9:1 in reproductive-age adults, alongside Sjögren’s syndrome (9:1) and autoimmune thyroid diseases (7:1) [8,9].

Historically, the conventional understanding attributed this dichotomy to a static hormonal and genetic framework. It is well established that the X chromosome, which houses the genome’s largest density of immune-related genes, including TLR7, Foxp3, CD40L, and CXCR3, imposes a unique gene dosage burden on females [10]. Although X-chromosome inactivation (XCI) theoretically equalizes gene expression between XX females and XY males, this process is incomplete. Approximately 15–23% of X-linked genes escape inactivation in human immune cells, leading to a double dosage of critical inflammatory regulators [11]. Superimposed on this genetic landscape is the endocrine modulation by sex hormones, where estrogens generally enhance humoral and cell-mediated immunity, while androgens exert suppressive effects [12].

However, the incomplete concordance of SLE in monozygotic twins (25–70%) and the profound sex divergence in familial risk—where daughters of affected mothers face a 1:40 risk compared to 1:250 for sons—underscore that intrinsic genetic templates alone cannot account for disease pathogenesis [10,11,12]. This epidemiological gap points to a critical dependency on environmental inputs, suggesting that the female immune system exists in a poised state where microbiota-derived metabolic signals act as the obligatory extrinsic stabilizers preventing the breach of tolerance.

We posit that the female immune system faces a unique evolutionary stability-plasticity dilemma. Unlike the male immune system, which is optimized for constant vigilance, the female system must possess the unique capacity to accommodate the extensive physiological remodeling of pregnancy. This requires the Regulatory T cell (Treg) network to maintain high intrinsic plasticity—the ability to dynamically expand and functionally adapt to tolerate the semi-allogeneic fetus [1]. Yet, this necessary flexibility renders the system structurally vulnerable. A Treg population that is plastic enough to acquire tolerance towards fetal antigens is also inherently unstable enough to lose Foxp3 expression and convert into pathogenic effector cells (ex-Tregs) under inflammatory duress [13].

This intrinsic instability was underscored by seminal research in 2024 [14]. Dou et al. revealed that the Xist ribonucleoprotein (RNP) complex, the fundamental machinery responsible for silencing the extra X chromosome, functions as a significant and female-exclusive autoantigen. Upon cell death, these complexes are released and constitutively sensitize innate sensors (TLR7/9), creating a state of autoimmune priming. This finding implies that healthy female immunity exists in a poised state, operating with a hair-trigger sensitivity to self-antigens. This raises a fundamental biological question: How has the female immune system successfully navigated millions of years of evolution without succumbing to spontaneous autoimmunity, given this constitutive immune priming?

We propose the “Metabolic Calibration Hypothesis”. We argue that the female immune system did not evolve in isolation but co-evolved with a complex community of commensal microbes. To counterbalance the intrinsic accelerator provided by X-linked genes and hormonal plasticity, the host outsourced the necessary extrinsic braking mechanisms to the microbiota. Microbial metabolites, particularly short-chain fatty acids (SCFAs) such as butyrate and reproductive tract metabolites like L-lactate, function as essential epigenetic and metabolic rheostats [15]. They function by inhibiting histone deacetylases (HDACs) or driving histone lactylation, thereby reinforcing the epigenetic stability of plastic Tregs and maintaining their suppressive lineage fidelity [16,17].

**Figure 1:**
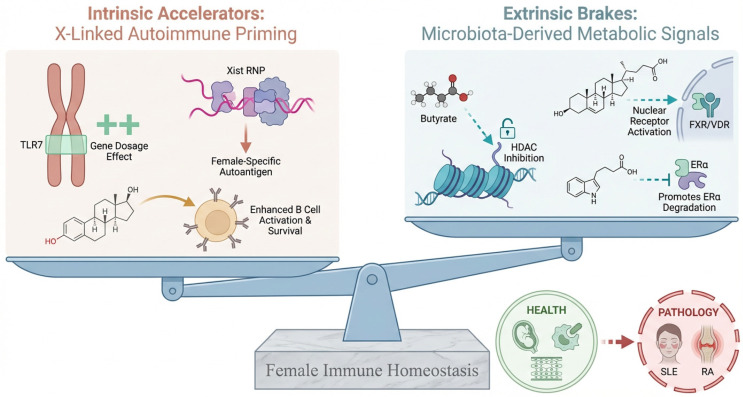
The Metabolic Calibration Hypothesis of Female Immunity. Female immune homeostasis is presented as a balance between intrinsic pro-inflammatory accelerators (e.g., Xist RNP, TLR7, estrogen) and extrinsic metabolic brakes derived from the microbiota (e.g., butyrate, secondary bile acids, indole derivatives). A calibrated state, where microbial metabolites successfully counteract the intrinsic accelerators, maintains health. An imbalance, caused by deficient metabolic braking, results in the over-reactivity that drives autoimmune pathology.

Within this conceptual framework, female autoimmune susceptibility is interpreted as a consequence of the evolutionary mismatch between a highly reactive, X-linked genetic architecture and the reduced metabolic outputs of the contemporary microbiome. To elucidate this “Host-Microbiota Metabolic Integration,” we conducted a narrative review of literature published primarily between January 2000 and February 2026, identifying relevant studies via PubMed, Web of Science, and Scopus. We utilized keywords including Xist RNP, Female Autoimmunity, Microbiota Metabolites, and Histone Lactylation, specifically prioritizing emerging mechanistic evidence from 2024 and 2026. Our search focused on the convergence of X-linked immune priming and microbial endocrinology; particular emphasis is placed on the mechanistic insights published in recent years. This review systematically evaluates these dynamics across several chapters. Following an analysis of the molecular interplay between Xist RNPs and microbial metabolites in Section 2, the subsequent chapters detail specific epigenetic and receptor-mediated pathways, including histone lactylation and bile acid signaling (Section 3 and Section 4). The broader physiological implications of this metabolic calibration, ranging from maternal-fetal tolerance to oncogenic co-option, are explored in Section 5. The manuscript concludes in Section 6 by defining Sexual Dimorphic Metabotyping as a foundation for precision medicine. This integrated model is visually summarized as a homeostatic equilibrium between intrinsic stimulatory factors and extrinsic regulatory signals (Figure 1).

## 2. Why Female Tregs Need Metabolic Support: The Molecular Confrontation Between Xist and Metabolites

Traditional models of sexual dimorphism have primarily focused on the duality of sex hormones and gene dosage. However, this framework fails to explain why the female immune system, despite its heightened antiviral competence, exhibits increased susceptibility to autoimmunity. Emerging evidence from 2024 and 2025 has fundamentally shifted this narrative from a hormonal imbalance to a molecular confrontation. We now understand that the female immune landscape is defined by a dynamic interaction: the inevitable accumulation of Xist ribonucleoprotein (RNP) complexes, which act as intrinsic accelerators sensitizing innate sensors, versus the extrinsic brakes provided by microbiota-derived metabolites [18,19].

This section reframes host susceptibility by proposing that female Regulatory T cells (Tregs) are not merely plastic by design, but are metabolically dependent. They require constant epigenetic locking by metabolites like butyrate to counteract the autoimmune priming driven by Xist. This dynamic equilibrium—where metabolic inputs actively raise the activation threshold that Xist lowers—constitutes the true biological basis of female immune balance [20].

### 2.1. The Intrinsic Accelerator: Xist RNPs and the TLR7/9 Hypersensitivity Loop

For decades, X-chromosome inactivation (XCI) was viewed largely as a gene dosage compensation mechanism. The lncRNA Xist coats one X chromosome to silence it. However, a landmark study by Dou et al. revealed a detrimental aspect to this essential biological process: the Xist RNA itself, along with its 81 associated binding proteins (RBPs), forms supramolecular complexes that are potent, female-specific autoantigens [21,22].

Unlike male cells, which lack Xist expression, female immune cells are burdened with the continuous management of these RNP complexes. Under physiological conditions, these complexes are sequestered. However, during the naturally high rates of lymphocyte turnover or in response to minor tissue injury, these complexes are released. Crucially, the Xist RNP complex does not merely act as an inert target for autoantibodies; it functions as a catalytic adjuvant. It specifically engages Toll-like Receptor 7 (TLR7) and TLR9 in plasmacytoid dendritic cells (pDCs) and B cells [23,24,25].

This engagement is not trivial. TLR7 itself is an X-linked gene that frequently escapes inactivation, leading to higher protein levels in females [11,26]. The synergy between elevated TLR7 dosage and the presence of its ligand (Xist RNA complexed with nucleic acids) creates a feed-forward inflammatory loop. This results in a constitutively elevated Interferon Signature (Type I IFN) in females, a phenotype previously termed molecular priming [27].

Therefore, the baseline state of the female immune system is not resting but poised. The threshold for triggering an autoimmune cascade is structurally lowered by the very mechanism (XCI) that defines female cellular identity. This creates a biological imperative: to prevent this heightened sensitivity from causing spontaneous autoimmunity, the system requires a powerful, constitutive suppressive force—a role that falls to the Treg network [28].

### 2.2. The Fragility of Female Tregs: Plasticity at the Cost of Stability

To counteract the Xist-driven interferon bias, female Tregs must exert robust suppression. However, the female reproductive imperative imposes a conflicting demand: Plasticity. Unlike the male Treg pool, which prioritizes rigid lineage stability for self-surveillance, female Tregs must retain the capacity to dedifferentiate or alter their functional state to accommodate the dynamic antigenic load of the menstrual cycle and, ultimately, the semi-allogeneic fetus [5,12].

This plasticity is encoded epigenetically at the Foxp3 locus. The stability of Foxp3 expression depends on the methylation status of the Conserved Non-coding Sequence 2 (CNS2), also known as the Treg-Specific Demethylated Region (TSDR) [29,30,31]. Recent high-resolution chromatin profiling suggests that female Tregs exhibit a higher degree of epigenetic heterogeneity at this locus compared to males [32]. This is partly due to the mosaicism of X-inactivation. Since Foxp3 is X-linked, female Tregs represent a mosaic population expressing either the maternal or paternal allele. While this diversity broadens the repertoire of antigen recognition, it introduces a structural fragility [33].

Under the high Type I IFN environment driven by Xist/TLR7 signaling, this fragility is exposed. Interferons activate the STAT1 signaling pathway, which can physically displace STAT5 (the stabilizer of Foxp3) from the Foxp3 promoter [34]. Consequently, in the absence of external stabilization, female Tregs are prone to losing Foxp3 expression and converting into pathogenic ex-Tregs (often Th17-like or IFNγ-producing Th1-like cells) [34]. This conversion essentially flips the immune regulator into an immune aggressor, a phenomenon central to the pathogenesis of SLE and Sjogren’s syndrome [27].

### 2.3. The Extrinsic Brake: Metabolic Epigenetics as the Counterweight

Here lies the crux of the Metabolic Calibration Hypothesis. Evolution has solved the problem of Xist-driven instability not by encoding more suppressor genes, but by coupling female immune stability to microbial metabolism. The gut microbiota acts as the extrinsic brake that counteracts the intrinsic accelerator.

A major molecular mediator in this interaction is Butyrate, a short-chain fatty acid (SCFA) produced by clostridial clusters. The mechanism is precise and antagonistic to the risk factors described above. Butyrate acts as a potent inhibitor of Class I histone deacetylases (HDACs), specifically HDAC3 [35,36].

In the context of the Xist-primed female immune system, Butyrate’s role is functionally significant:

Chromatin Locking at CNS1: While Xist/TLR7 signaling may destabilize the Treg lineage via inflammatory cytokines, Butyrate promotes the hyperacetylation of Histone H3 Lysine 27 (H3K27ac) at the Foxp3 CNS1 enhancer [37]. This epigenetic mark facilitates a molecular lock, keeping the chromatin open for the binding of stabilizing transcription factors like Smad3 and NFAT, even in the presence of inflammatory perturbations [38].

Threshold Elevation: By supporting high Foxp3 expression, Butyrate effectively raises the activation threshold of the immune system. It may desensitize dendritic cells to the low-grade stimulation provided by Xist RNPs, thereby raising the threshold against autoimmune priming [39].

Direct Antagonism of the Interferon Signature: Beyond Tregs, recent data suggests that Butyrate can directly suppress the TLR7 signaling pathway in B cells by downregulating the expression of Irf7, the master regulator of the interferon response [40].

Conceptually, this suggests that the health of the female immune system benefits from a continuous supply of Butyrate to maintain this molecular standoff. The risk is endogenous and constant (Xist); the protection is exogenous and variable (diet/microbiome) [41,42].

### 2.4. Dysregulation of Metabolic Calibration: A Context-Dependent Risk Factor

When viewing female autoimmunity through this lens, disease susceptibility involves a disruption of metabolic calibration that exacerbates intrinsic genetic risks. However, translating this mechanistic insight into clinical causality requires a nuanced understanding of disease heterogeneity.

While meta-analyses in patients with established SLE and RA frequently report a depletion of butyrate-producing genera such as *Faecalibacterium* and *Roseburia* [43,44], this pattern is not universal. Recent longitudinal studies indicate that dysbiosis may be less pronounced in early-stage, treatment-naive patients compared to those with long-standing disease, suggesting that microbiome shifts could be partially driven by chronic inflammation or immunosuppressive medications [45,46]. Furthermore, the impact of SCFA deficiency appears uneven across autoimmune spectrums; for instance, while strongly correlated with disease activity in SLE and RA, the association is more variable in Multiple Sclerosis, where other metabolites (e.g., tryptophan derivatives) may play a more dominant role [47,48].

Despite these variations, the mechanistic consequence of butyrate deficiency—where it occurs—remains critical for female immune stability. In the absence of sufficient HDAC inhibition:

The Foxp3 CNS1 enhancer is prone to deacetylation and reduced accessibility [49].

Treg plasticity may shift toward instability (ex-Treg formation) under inflammatory conditions [50].

The unbuffered Xist/TLR7 signaling in B cells and pDCs promotes a robust Type I Interferon response [51].

Therefore, rather than viewing dysbiosis as the sole trigger, we propose that the “Western Diet” (low fiber, low SCFA production) acts as a variable environmental modifier. It disproportionately affects females by unmasking an evolutionarily conserved X-linked vulnerability [52,53]. This is supported by recent single-cell transcriptomics from 2025, which identified a subset of “Metabolically Starved Tregs” in the synovial fluid of female RA patients characterized by epigenetic erosion, validating this failure of metabolic support in specific tissue microenvironments.

**Figure 2:**
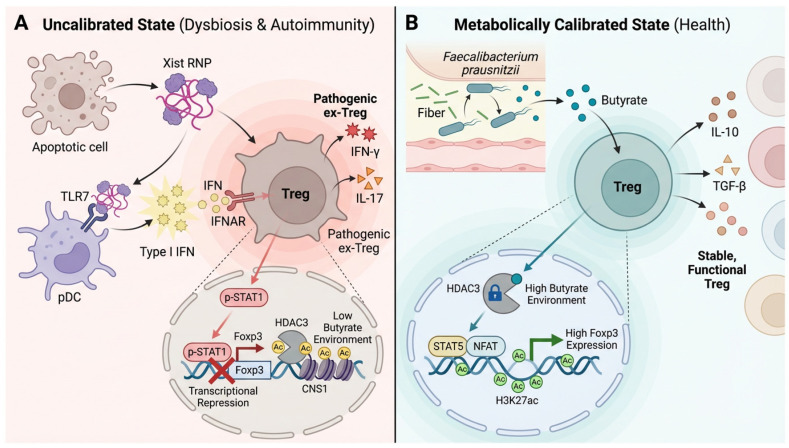
Molecular Control of Treg Stability by Microbial Metabolites. (**A**) In an uncalibrated state, TLR7-mediated sensing of Xist RNP triggers IFN-I signaling that represses Foxp3. Concurrently, butyrate deficiency permits HDAC3 to remove the activating H3K27ac mark at the Foxp3-CNS1 enhancer. This two-hit mechanism destabilizes Treg identity, promoting their conversion into pathogenic, inflammatory ex-Tregs. (**B**) In a calibrated state, microbiota-derived butyrate directly inhibits HDAC3, thereby preserving the H3K27ac landscape at the Foxp3-CNS1 locus. This epigenetic protection ensures stable Foxp3 expression and maintains the suppressive function of the Treg lineage.

In summary, female Tregs do not function in a vacuum. They are metabolically dependent to microbial signals. The high biological cost of the X chromosome—specifically the immunogenicity of Xist—necessitates a compensatory mechanism. Microbiota-derived metabolites provide this support, transforming a genetically vulnerable system into a robustly plastic one. The loss of this metabolic support is the second hit that transforms genetic susceptibility into clinical autoimmunity. This molecular confrontation, where microbiota-derived butyrate provides a critical epigenetic countermeasure to the intrinsic instability imposed by the Xist/TLR7/IFN axis on female Tregs, is mechanistically depicted in Figure 2.

## 3. Metabolic-Epigenetic Stabilization: Counteracting X-Linked Genomic Instability

The female immune system operates under a unique genomic tension. As elucidated in the preceding sections, the interplay between the female-specific Xist ribonucleoprotein (RNP) complex, the gene dosage escape of TLR7, and the heightened activity of the X-linked histone demethylase Kdm6a (which encodes the protein UTX) creates an intrinsic chromatin landscape that is constitutively poised for inflammatory transcription [24]. While this epigenetic architecture confers superior antiviral resistance, it renders the Regulatory T cell (Treg) lineage structurally vulnerable to lineage washout, whereby Tregs lose Foxp3 expression and transdifferentiate into effector phenotypes under stress [54]. We propose the Metabolic Calibration Hypothesis, positing that the female host has co-evolved to obligatorily rely on microbial metabolites as extrinsic epigenetic anchors. By acting as substrates or inhibitors for chromatin-modifying enzymes, these metabolites impose the necessary epigenetic rigidity to counteract intrinsic X-linked stimulatory signals.

### 3.1. The Short-Chain Fatty Acid Axis: Systemic Epigenetic Locking via HDAC Inhibition

Short-chain fatty acids (SCFAs), primarily acetate, propionate, and butyrate, are the canonical fermentation products of dietary fiber by colonic commensals such as *Faecalibacterium prausnitzii* and *Eubacterium rectale* [15,55,56]. In the context of female immunity, their function transcends mere energy provision; they serve as critical epigenetic rheostats that enforce Treg stability against the destabilizing forces of hormonal fluctuation and X-linked priming.

#### 3.1.1. Mechanism: Epigenetic Locking of the CNS1 Enhancer

The primary mechanism by which butyrate stabilizes the fragile female Treg network is through the potent, non-competitive inhibition of Class I Histone Deacetylases (specifically HDAC1 and HDAC3) [57,58]. In female-biased autoimmune conditions like SLE, where Xist RNPs constitutively ligate TLR7 to drive type I interferon and IL-6 production, Tregs are under immense pressure to lose their identity and convert into pathogenic Th17-like cells [59]. Butyrate functions as a molecular brake on this conversion. By inhibiting HDACs, butyrate prevents the deacetylation of Histone H3 Lysine 27 (H3K27ac) at the Foxp3 promoter and, most critically, at the Conserved Non-coding Sequence 1 (CNS1) enhancer element [35,60,61].

The CNS1 region acts as a sensor for microbial signals and is indispensable for the extrathymic generation of peripheral Tregs (pTregs), the specific subset responsible for maintaining maternal-fetal tolerance and preventing mucosal inflammation [62]. Since pTregs are inherently more plastic and susceptible to environmental flux than their thymic counterparts, the specific epigenetic locking of CNS1 by microbial butyrate is evolutionarily essential for female [63]. Mechanistic studies utilizing ATAC-seq and ChIP-seq have demonstrated that in the absence of butyrate, the CNS1 locus in pTregs becomes rapidly methylated and inaccessible, a process significantly accelerated by the inflammatory cytokine milieu typical of female autoimmunity [64]. Thus, butyrate provides the necessary tonic signal to keep the master switch of tolerance accessible, counterbalancing the intrinsic genetic drive toward chromatin remodeling and inflammation.

#### 3.1.2. Sexual Dimorphism in SCFA Sensing: The Estrogen-Transporter Axis

Crucially, the efficacy of this epigenetic stabilization is strictly gated by host hormones, revealing a novel and underappreciated layer of sexual dimorphism [65]. The entry of butyrate and propionate into immune cells and colonic epithelia is not passive but mediated by specific transporters, primarily MCT1 (Monocarboxylate Transporter 1) and SMCT1 (Sodium-coupled Monocarboxylate Transporter 1, encoded by Slc5a8). Recent transcriptomic analyses have revealed that the expression of these transporters is sexually dimorphic and directly modulated by Estrogen Receptor alpha (ERα) signaling [29,66].

In murine models of intestinal inflammation, high physiological levels of estradiol (E2), typical of the late follicular phase, have been observed to transiently downregulate Slc5a8 and Mct1 expression in specific murine models of intestinal inflammation [67]. In these models, this downregulation creates a metabolic window of vulnerability: during peak estrogen, even if the microbiota produces adequate levels of butyrate, the host’s ability to uptake this epigenetic stabilizer is compromised [68]. While human validation is pending, these preclinical data generate a compelling hypothesis: females might biologically require a higher luminal concentration of SCFAs than males to achieve the intracellular levels necessary for effective HDAC inhibition during specific cycle phases [66,69,70,71]. Consequently, rather than applying one-size-fits-all probiotic dosing strategies, future translational research must determine whether metabotyping should account not just for microbial production capacity, but for the host’s sex-specific and cycle-dependent sensing and transport efficiency.

### 3.2. The Lactate-Lactylation Axis: Metabolic Reprogramming of the Reproductive Tract

While SCFAs dominate the gut-systemic axis, the female reproductive tract (FRT) constitutes a highly specialized metabolic niche. In healthy reproductive-age women, the vaginal microbiota is dominated by *Lactobacillus* species (e.g., *L. crispatus*, *L. jensenii*), which ferment glycogen to maintain L-lactate concentrations at supraphysiological levels (~110 mM), vastly exceeding serum levels (~1–2 mM) [72,73]. Until recently, this lactate was historically recognized primarily as a pH buffer. However, landmark studies published between 2024 and 2025 have redefined lactate as a significant epigenetic regulator that drives Histone Lactylation (Kla), a modification that fundamentally rewires T cell fate [74,75,76].

#### 3.2.1. The Biochemistry of Histone Lactylation (H3K18la)

Histone lactylation represents a direct metabolic-epigenetic link where intracellular lactate is converted to lactyl-CoA by the enzyme Acetyl-CoA Synthetase 2 (ACSS2) [77,78]. A pivotal 2025 study in Cell Metabolism definitively characterized the kinetics of this reaction, demonstrating that ACSS2 acts as a lactate sensor that is upregulated and translocated to the nucleus under high-lactate conditions. The histone acetyltransferase p300 (also known as KAT2B) then utilizes the generated lactyl-CoA as a substrate to add lactyl groups to lysine residues on histone tails, most notably generating Histone H3 Lysine 18 lactylation (H3K18la) [74,79]. In the FRT, the sustained high-lactate microenvironment forces a profound shift in the epigenetic landscape of infiltrating CD4+ T cells [75]. Unlike acetylation, which is kinetically rapid and transient, lactylation accumulates more slowly and serves as a metabolic scar—a long-term memory of the metabolic environment that persists even after the signal is removed [80]. Recent chromatin immunoprecipitation sequencing (ChIP-seq) data demonstrates that H3K18la is preferentially enriched at the promoters of stem-like and regulatory genes, including Foxp3, Il10, and Tgfb1, while being notably absent from the promoters of terminal effector genes like Ifng and Gzmb [81].

#### 3.2.2. Direct Reprogramming of the Th17/Treg Balance

This mechanism acts as the critical determinant for the female immune system’s stability-plasticity dilemma [82]. The FRT must vigorously defend against sexually transmitted pathogens (requiring Th17 cells) while simultaneously tolerating sperm and the semi-allogeneic fetus (requiring Tregs). Histone lactylation solves this by promoting plasticity. High levels of H3K18la at the Foxp3 locus allow T cells to maintain regulatory function even in the presence of pro-inflammatory cytokines like IL-6, which would normally drive them toward a pathogenic Th17 phenotype [83]. Essentially, the microbiota-derived lactate lactylates the genome of local T cells, rendering them resistant to autoimmune conversion [81,84]. This explains why a loss of *Lactobacillus* dominance (as seen in Bacterial Vaginosis) is immunologically catastrophic: the disappearance of lactate leads to a rapid loss of the H3K18la mark, causing mucosal Tregs to lose their metabolic shield and revert to an inflammatory state, thereby opening the door to HIV acquisition and adverse pregnancy outcomes [80,85].

#### 3.2.3. Mitochondrial Fueling via MGAT1: A Metabolic Checkpoint

Beyond epigenetics, lactate supports the metabolic demands of female Tregs through a distinct mitochondrial pathway. Unlike effector T cells, which rely on aerobic glycolysis and are often functionally paralyzed by high lactate, Tregs in the FRT possess a unique metabolic adaptation: the upregulation of N-acetylglucosaminyltransferase I (MGAT1) [86]. A breakthrough 2024 study revealed that lactate feeds into the TCA cycle to support MGAT1-dependent N-glycosylation of key surface receptors (e.g., CD25, CTLA-4) [86]. This specific glycosylation pattern prevents the endocytosis and lysosomal degradation of these receptors, ensuring their surface retention [54]. Thus, vaginal lactate creates a metabolic-proteomic reinforcement loop: it ensures that Tregs not only transcribe tolerance genes (via lactylation) but also physically retain the surface machinery necessary to execute suppression. This dual regulation highlights why the *Lactobacillus*-dominated microbiota is non-redundant for female health.

## 4. Receptor Crosstalk: Sex Hormone-Metabolite Integration

The second dimension of the Metabolic Calibration Hypothesis involves the direct physical and functional crosstalk between microbial metabolite sensors (Nuclear Receptors) and sex hormone receptors (ER and PR). This integration explains why the female immune system is hypersensitive to specific metabolic deficits, creating a molecular antagonism between microbial ligands and host hormones [5].

### 4.1. The Bile Acid—Nuclear Receptor Axis: The Estrogen Bottleneck

Secondary bile acids (BAs), such as lithocholic acid (LCA), 3-oxoLCA, and isoalloLCA, are generated solely by the microbiota (specifically *Clostridiales* species like *Clostridium scindens*) via 7α-dehydroxylation [87]. These molecules are potent ligands for the Vitamin D Receptor (VDR), the Farnesoid X Receptor (FXR), and modulators of the orphan receptor NR4A1 (Nur77) [82]. Consistent with this, gut microbiota-derived secondary bile acids have been shown to activate FXR and downstream incretin pathways, underscoring their role as metabolic hormones in host physiology [88].

#### 4.1.1. The Mechanism of Treg Induction

Secondary BAs function as metabolic hormones. Beyond the NR4A1 pathway, secondary BAs such as isoDCA (3β-hydroxydeoxycholic acid) exert their immunomodulatory effects by targeting dendritic cells (DCs) via the Farnesoid X Receptor (FXR) [89]. IsoalloLCA, for instance, triggers the formation of a mitochondrial-associated complex involving NR4A1, which subsequently translocates to the nucleus to bind the Foxp3 CNS3 enhancer (the pioneer element). This binding increases chromatin accessibility, facilitating the recruitment of other transcription factors and enhancing Treg differentiation [90]. Concurrently, 3-oxoLCA functions as an inverse agonist of RORγt, directly suppressing the differentiation of Th17 cells [91]. Together, these metabolites enforce a dominant Treg phenotype in the lamina propria, which is critical for systemic tolerance.

#### 4.1.2. The Estrogen Bottleneck in Bile Acid Synthesis

The sexual dimorphism in this axis is profound and structural. The synthesis of primary bile acids in the liver is regulated by enzymes such as Cyp7a1, Cyp8b1, and Cyp7b1. Crucially, estrogen (E2) is a potent repressor of Cyp7b1 (oxysterol 7α-hydroxylase), a key enzyme in the alternative bile acid synthesis pathway [92]. Consequently, females naturally possess a smaller and compositionally distinct bile acid pool compared to males [93]. Indeed, genetic defects in CYP7B1 provide direct evidence for the critical role of this enzyme: patients with congenital bile acid synthesis disorder type 3 (BASD3) due to CYP7b1 deficiency present with neonatal cholestasis, hepatomegaly, markedly elevated direct bilirubin and aminotransferases, but normal γ-glutamyltransferase (GGT). Without timely replacement therapy with chenodeoxycholic acid (CDCA), the condition rapidly progresses to liver failure and death [94]. This human loss-of-function model underscores how estrogen-mediated repression of Cyp7b1 physiologically constrains the bile acid pool in females.

This creates an Estrogen Bottleneck: because the starting pool of primary BAs is limited by estrogen, females are disproportionately dependent on the efficiency of the gut microbiota to convert this limited pool into immunomodulatory secondary BAs [95]. A 20% reduction in BA-transforming bacteria might be tolerable in males (who have a surplus substrate pool) but could be deleterious in females, dropping secondary BA levels below the threshold (Kd) required for NR4A1 or VDR activation [96]. This metabolic threshold provides a mechanistic explanation for why gut dysbiosis triggers autoimmune flares more readily in women: their metabolic margin of error is narrower due to hormonal constraints on substrate availability [97]. Clinical observations in Multiple Sclerosis (MS) confirm that female patients exhibit a more severe depletion of secondary BAs than male patients, correlating with higher disease activity [98]. Furthermore, recent findings suggest this deficit specifically impairs the VDR-dependent maintenance of intestinal barrier integrity in females. Using an experimental colitis model, Liu et al. demonstrated that gut epithelial VDR signaling is essential for suppressing epithelial cell apoptosis and maintaining barrier integrity; loss of epithelial VDR leads to microbiota-driven mucosal inflammation, a mechanism highly relevant to female-biased inflammatory bowel diseases [99,100].

### 4.2. The Tryptophan-AhR-Estrogen Axis: A Molecular Antagonism

Tryptophan metabolism represents the most direct example of Molecular Antagonism between the microbiome and the endocrine system. Gut commensals convert dietary tryptophan into indole derivatives (e.g., indole-3-propionic acid (IPA), indole-3-aldehyde (IAld), indole-3-lactic acid (ILA)), which act as high-affinity ligands for the Aryl Hydrocarbon Receptor (AhR) [95,101,102].

#### 4.2.1. AhR as a Ubiquitin Ligase for ERα

While AhR activation promotes barrier integrity and Treg differentiation, its interaction with Estrogen Receptor alpha (ERα) is defining for female immunity [103,104]. Ligand-bound AhR physically associates with ERα and the ubiquitin ligase CUL4B, assembling a proteasomal degradation complex [105]. Consistent with this, AhR activation in the ovary has been shown to regulate the expression of estrogen-metabolizing enzymes CYP1A1 and CYP1B1, which hydroxylate estradiol into distinct metabolites [106,107,108]. This positions AhR as a dual regulator of estrogen signaling: it not only promotes ERα degradation but also controls the metabolic clearance and bioactivation of estrogen itself. This means that microbial AhR ligands can actively drive the ubiquitin-mediated degradation of ERα, thereby dampening estrogen-driven inflammatory responses (e.g., the hyper-activation of B cells or Type I interferon production) [109]. During the follicular phase, when high E2 drives potentially pathogenic Th1/Th17 responses, the presence of microbial indoles serves as a metabolic anti-estrogen, buffering the system against hormonal overstimulation [110]. Conversely, in specific contexts like endometriosis or ER+ breast cancer, the loss of AhR ligands prevents this negative feedback, allowing ERα signaling to proceed unchecked [111,112]. This sexual dimorphism in immune response to microbial signals is evident even in early life, where preterm female neonates exhibit enhanced monocyte activation compared to males, correlating with their lower clinical risk of sepsis [113].

#### 4.2.2. The Indole-Deficit Phenotype in Autoimmunity

In female mice, dietary tryptophan depletion exacerbates central nervous system autoimmunity (EAE) significantly more than in males, a defect reversible by AhR ligand supplementation [114]. This suggests that the female immune system has evolved to rely on a tonic level of microbial indoles to counterbalance the pro-stimulatory effects of estrogen [115]. When this metabolic brake fails (due to low-tryptophan diets or loss of indole-producing *Peptostreptococcus*), the intrinsic accelerator of estrogen takes over, driving the breakdown of tolerance. Furthermore, novel findings indicate that AhR ligands also modulate the expression of aromatase (CYP19A1) in peripheral tissues [116]. A recent study by Sharma et al. (2025) demonstrated that the potent AhR ligand TCDD selectively suppresses CYP19A1 expression via the proximal promoter PII in granulosa cells, leading to reduced estradiol production—an effect reversed by pharmacological inhibition of AhR [117]. This suggests a bidirectional loop where microbial metabolites, acting through AhR, could locally tune estrogen synthesis itself [118]. This mechanism is further supported by evidence linking AhR activation to the suppression of pathogenic B cell differentiation in female-biased models of lupus [119,120].

### 4.3. Phytoestrogens: The Metabotype-Dependent ERβ Agonism

Finally, the metabolism of dietary isoflavones (phytoestrogens) illustrates the concept of Metabotyping and its critical relevance to female immunity. Soy-derived daidzein is biologically inert until it is converted into S-Equol by specific gut bacteria (e.g., *Slackia isoflavoniconvertens*, *Adlercreutzia equolifaciens*) [121].

Selective ERβ Activation and Barrier Defense S-Equol is a selective agonist for Estrogen Receptor beta (ERβ) with a high binding affinity (Ki ~ 0.73 nM), while having negligible activity at ERα [122]. In the gut and immune system, ERβ signaling is generally anti-inflammatory and barrier-protective, opposing the often pro-inflammatory ERα pathway. By activating ERβ, S-Equol inhibits the NF-κB inflammasome and enhances the expression of tight junction proteins (Claudin-5, Occludin) [118,123]. However, only 30–50% of the human population hosts the microbiota necessary to produce S-Equol (Equol-Producers) [124,125]. This dichotomous metabotype creates a stark divergence in immune regulation [126]. Non-producers are effectively deprived of a potent, diet-derived ERβ agonist. In the context of female autoimmunity, where the ERα/ERβ balance is often skewed toward ERα, the inability to produce Equol represents a missing metabolic lever to restore balance [127]. This highlights why generic dietary advice fails: a soy-rich diet will only immunologically benefit the specific subset of women who possess the requisite microbial enzymatic machinery. Recent population-level analyses have further stratified this effect, showing that Equol-producing women have significantly lower markers of systemic inflammation compared to non-producers, an effect not observed in males [128,129,130].

### 4.4. Synthesis: The Extrinsic Scaffold

In summary, the mechanisms detailed in Section 3 and Section 4 reveal a sophisticated system of Host-Microbiota Metabolic Integration. The female immune system does not operate in isolation; it utilizes:

Epigenetic Anchors (Butyrate/Lactate) to lock the unstable chromatin of plastic Tregs against X-linked priming [131]. Nuclear Receptor Ligands (Bile Acids) to overcome hormonal bottlenecks in immune regulation. Cross-Regulatory Signaling (Indoles/Equol) to directly dampen estrogenic overstimulation via receptor degradation or antagonism [132,133,134].

This integration implies that the reference range for these metabolites is not universal but sex-specific. Females, due to their unique genetic (Xist) and hormonal (E2) landscape, have a biologically higher requirement for these extrinsic brakes [135,136]. The failure to meet this metabolic demand, driven by antibiotic overuse, low-fiber diets, or the loss of key converter strains, disproportionately destabilizes female immunity [137]. This creates a Metabolic-Genetic Mismatch, precipitating the sex-biased disease spectrum observed clinically [138,139]. Understanding this mismatch is crucial for developing therapeutic interventions, as simply restoring microbial diversity may be insufficient without restoring the specific metabolic pathways that calibrate female immune plasticity [13]. Future therapies must essentially act as metabolic prosthetics, providing the ligands (like lactate or equol) that the dysbiotic female microbiome fails to produce [140,141].

## 5. Context-Dependent Plasticity: The Metabolic Checkpoints of Disease

The Host-Microbiota Metabolic Integration framework established in the preceding sections demonstrates that female immune homeostasis is not a static property but a dynamic equilibrium maintained by extrinsic microbial signals [142]. However, the functional outcome of this metabolic signaling is strictly context-dependent. Conserved metabolic mechanisms, including histone lactylation, HDAC inhibition, and nuclear receptor activation function as the guardian of physiological tolerance in pregnancy, the failed brake in autoimmunity, or the hijacked accomplice in malignancy [143]. This section dissects the divergent immunological outcomes driven by the bioavailability and functional co-option of microbial metabolites across four distinct female-specific contexts.

### 5.1. Autoimmune Diseases: The Failure of the Epigenetic Brake

In female-predominant autoimmune diseases such as Systemic Lupus Erythematosus (SLE) and Rheumatoid Arthritis (RA), the pathogenesis represents a Metabolic-Genetic Mismatch. As detailed in Section 2, the female immune system possesses an intrinsic genetic accelerator (Xist RNPs, TLR7 dosage, and Kdm6a activity) that lowers the activation threshold for innate signaling. In healthy states, this is counterbalanced by a metabolic brake (SCFA-mediated HDAC inhibition) [144]. In autoimmunity, this brake fails.

Recent multi-cohort metagenomic analyses published in 2025 have confirmed that SLE patients exhibit a specific functional dysbiosis characterized not just by taxonomic shifts, but by a collapse in the capacity to synthesize butyrate and secondary bile acids [40,145,146,147]. Specifically, there is a marked depletion of the Lachnospiraceae family (major butyrate producers) and a concurrent expansion of *Ruminococcus gnavus* (reclassified as *Mediterraneibacter gnavus*), a mucin-degrading pathobiont that produces inflammatory lipoglycans but lacks the gene clusters for butyrate synthesis [148,149]. The physiological consequence of this metabolic deficit is the epigenetic destabilization of peripheral Tregs. Without sufficient butyrate to inhibit HDACs, the Foxp3 CNS1 enhancer becomes inaccessible (deacetylated and methylated). Consequently, the plastic female Tregs, when exposed to chronic Xist-RNP autoantigens and high Type I interferons, lose their suppressive identity [150,151,152,153]. Single-cell ATAC-seq studies have revealed that these starved Tregs transdifferentiate into ex-Treg Th17-like cells (Foxp3^low^ IL-17^+^), which are pathogenic drivers of lupus nephritis [153,154]. Thus, autoimmunity is unleashed because the extrinsic metabolic signal required to lock the volatile female Treg lineage is absent [14].

Metabolic Checkpoint 1: The Butyrate-HDAC Brake

Status in Autoimmunity: Failed/Deficient.

Mechanism: Loss of commensal butyrate producers → Unchecked HDAC activity → Deacetylation of Foxp3 CNS1 → Loss of Treg lineage stability.

Outcome: The intrinsic X-linked accelerator (Xist/TLR7) proceeds unchecked, driving the conversion of plastic Tregs into pathogenic effectors [155].

### 5.2. Reproductive Tract Infections: Collapse of the Lactate Shield

In the female reproductive tract, homeostasis relies on the massive production of L-lactate by *Lactobacillus crispatus*, which maintains the Lactate-Lactylation axis described in Section 3.2 [156]. Bacterial Vaginosis (BV) represents a significant disruption of this local metabolic organ [157,158]. When protective Lactobacilli are displaced by anaerobes such as *Gardnerella vaginalis* and *Prevotella*, L-lactate concentrations plummet (from >100 mM to <20 mM), while succinate and short-chain fatty acids (like acetate) rise [159]. This metabolic shift is not merely a change in pH; it is a signaling failure. The loss of lactate halts the constitutive activation of GPR81 on dendritic cells and, more critically, depletes the substrate for Histone Lactylation (H3K18la) in resident T cells [160]. Without the metabolic scar of H3K18la to enforce their regulatory phenotype, mucosal Tregs lose their suppression capacity [161]. Concurrently, the rise in succinate acts as a danger signal, stabilizing HIF-1α in macrophages and driving an aggressive inflammatory response (IL-1β, TNF-α) [157]. This creates an environment conducive for viral acquisition: the loss of metabolic suppression recruits activated, HIV-susceptible CD4+ T cells to the mucosa. A 2024 prospective study demonstrated that women with a lactate-deficient cervicovaginal metabolome had a 3-fold higher risk of HIV acquisition, independent of barrier integrity, highlighting that metabolic signaling is a primary layer of mucosal defense [162].

Metabolic Checkpoint 2: The Lactate-Lactylation Shield

Status in Infection (BV): Collapsed.

Mechanism: Replacement of Lactobacilli with anaerobes → Depletion of L-Lactate → Loss of GPR81 signaling and H3K18la marks → Succinate-driven inflammation.

Outcome: Disruption of the poised tolerogenic state, leading to mucosal inflammation and recruitment of HIV targets.

### 5.3. Gynecologic Malignancies: The Warburg Mimicry Trap

Conversely, in gynecologic malignancies such as high-grade serous ovarian cancer (HGSOC) and cervical squamous cell carcinoma, the immune system faces the opposite problem: pathological tolerance. Here, we argue that tumors actively hijack the metabolic mechanisms evolved for pregnancy. Tumor cells exhibit the Warburg effect, producing massive quantities of lactate, creating a microenvironment that mimics the high-lactate niche of the healthy vagina or the pregnant uterus [163,164]. While high lactate paralyzes effector CD8+ T cells (by inhibiting their glycolytic flux), Regulatory T cells are metabolically privileged. Tumoral Tregs avidly uptake lactate via the transporter MCT1 (SLC16A1) and use it to fuel oxidative phosphorylation (OXPHOS), sustaining their survival in the hypoxic niche [165,166,167]. More importantly, recent evidence from 2025–2026 reveals that tumors exploit the Histone Lactylation pathway. The high intratumoral lactate load drives hyper-lactylation (H3K18la) at the promoters of Foxp3, CTLA4, and ICOS in infiltrating Tregs. This chemically locks these cells into a Super-Treg phenotype with enhanced suppressive potency [167]. Essentially, tumors may evade the immune system by mimicking a fetus or commensal organism to function as an entity protected by a high-lactate shield. This effect is reinforced by intratumoral bacteria such as *Fusobacterium nucleatum* in cervical cancer, which produce butyrate locally to synergize with lactate and epigenetically silence anti-tumor immunity [168]. This Warburg Mimicry explains why checkpoint inhibitors often fail in cold gynecologic tumors: the immune exclusion is reinforced by the very metabolic signals (lactate/SCFA) that the host naturally uses to maintain tolerance [169].

Metabolic Checkpoint 3: The Warburg Mimicry Mechanism

Status in Cancer: Hijacked/Hyperactive. Mechanism: Tumor-derived lactate mimics the reproductive tract microenvironment → MCT1-mediated uptake by Tregs → Excessive H3K18la deposition → Formation of Super-Tregs.

Outcome: Malignant cells co-opt the physiological Lactate-Treg axis evolved for pregnancy to enforce pathological immune evasion.

### 5.4. Pregnancy: The Physiological Calibration

**Figure 3:**
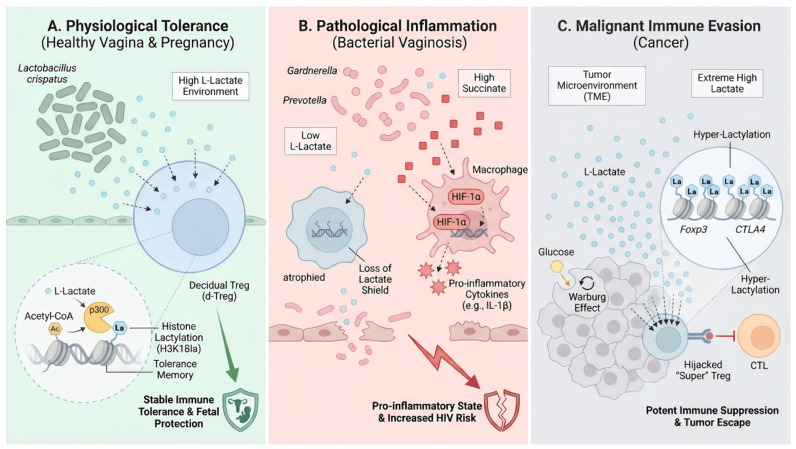
Context-Dependent Immunomodulatory Roles of Lactate. (**A**) Physiological Tolerance. In healthy mucosa, *Lactobacillus*-derived lactate promotes H3K18 lactylation (H3K18la) in Tregs, epigenetically reinforcing their stable, tolerogenic function. (**B**) Pathological Inflammation. During dysbiosis, the loss of the lactate shield and accumulation of succinate destabilizes Tregs and promotes macrophage-driven inflammation. (**C**) Malignant Immune Evasion. In the tumor microenvironment, cancer-secreted lactate drives hyper-lactylation of key immunosuppressive genes in Tregs, hijacking them into a super-suppressive phenotype that facilitates tumor escape.

Pregnancy represents a critical state of Metabolic Calibration, where systemic and local metabolic signals coordinate to solve the ultimate immunological challenge: tolerating a semi-allogeneic fetus while maintaining pathogen defense. During gestation, the host orchestrates a systemic metabolic remodeling. The maternal gut microbiome undergoes a progressive shift (resembling metabolic syndrome) to maximize energy extraction, leading to increased circulating levels of SCFAs [170,171]. These SCFAs act systemically to expand the pTreg pool via HDAC inhibition [172]. Simultaneously, the vaginal microbiome becomes less diverse and strictly dominated by *Lactobacillus* species (especially *L. crispatus*), ensuring maximal lactate production [157,162]. This dual metabolic surge creates a systemic-local tolerance loop. Systemic butyrate primes the Treg pool, while local lactate in the reproductive tract chemically modifies (via lactylation) the chromatin of Tregs specifically recruited to the decidua [173]. Recent data indicates that decidual Tregs exhibit a unique metabolic imprint characterized by high H3K18la and MGAT1 activity, which renders them resistant to the inflammatory signals of parturition until term [174]. In pathologies like preeclampsia, this axis fractures: a reduction in butyrate-producing bacteria and a loss of vaginal *Lactobacillus* dominance correlate with defective Treg function and placental inflammation [175,176]. Thus, pregnancy is the successful execution of the metabolic stability program that fails in autoimmunity and is mimicked in cancer. The starkly contrasting roles of a single metabolite, lactate, across these different female-specific contexts—acting as a physiological shield, a marker of pathological failure, or a hijacked weapon—powerfully illustrate this principle of context-dependent plasticity (Figure 3).

Metabolic Checkpoint 4: The Physiological Calibration

Status in Pregnancy: Optimized/Coordinated.

Mechanism: Systemic surge in gut SCFAs + Maximal vaginal L-Lactate → Synergistic epigenetic stabilization (Acetylation + Lactylation) of decidual Tregs.

Outcome: A robust, metabolically enforced tolerance network that protects the fetus while preventing infection, balancing the stability-plasticity trade-off.

## 6. Challenges and Limitations in Translational Research: From One-Size-Fits-All to Sexual Dimorphic Metabotyping

The Host-Microbiota Metabolic Integration framework established in this review provides a mechanistic roadmap for understanding the sex-biased nature of immune dysregulation. However, the translation of these insights into clinical practice is currently stalled by limitations in experimental reducibility [177]. Despite the theoretical promise of microbiome therapeutics, recent high-profile clinical trials—such as the use of generic *Lactobacillus* probiotics for preventing recurrent bacterial vaginosis or fecal microbiota transplantation (FMT) for SLE—have yielded inconsistent or underwhelming results [178,179,180]. We argue that these failures do not invalidate the biological premise but rather highlight a fundamental flaw in the current clinical paradigm: the lack of personalization and the disregard for sexual dimorphism [181,182,183]. However, we emphasize that this framework is currently theoretical. Moving from correlation to clinical diagnostics requires overcoming several critical hurdles: (1) demonstrating reproducibility of metabolite thresholds across diverse human cohorts; (2) differentiating tissue-specific metabolic effects from systemic serum levels; and (3) accounting for confounding factors such as medication use (e.g., immunosuppressants), dietary heterogeneity, and circadian rhythmicity.

The prevailing strategy of administering standardized bacterial consortia fails to account for the host’s intrinsic metabolic competence and hormonal context. Addressing sexual dimorphism in metabotyping should become a priority for future research. This precision medicine approach stratifies female patients based on their ‘Holobiont Potential,’ which integrates the microbiome’s capacity to produce immunomodulatory metabolites with the host tissue’s ability to sense them, rather than relying solely on disease activity indices like SLEDAI. Here, we define the critical challenges and propose three clinically actionable metabotypes and a novel precision window strategy for intervention.

### 6.1. The Causality and Model Fidelity Challenge

A primary obstacle in translating metabolic findings is the causality loop. While cross-sectional studies confirm that SLE patients lack butyrate-producing Lachnospiraceae [184,185]. it remains technically difficult to distinguish whether this dysbiosis is a driver of autoimmunity or a secondary consequence of the inflammatory milieu (e.g., high oxidative stress depleting anaerobes). Recent advances utilizing bi-directional Mendelian Randomization have begun to clarify this, suggesting a causal effect of specific SCFA-producing taxa on reduced SLE risk, yet human longitudinal validation is sparse [186,187].

Furthermore, the sex gap in preclinical modeling remains pervasive. Standard laboratory mice are naturally robust Equol-Producers (unlike humans) and do not experience a menstrual cycle comparable to the human follicular-luteal transition. This renders murine models poor predictors of how hormonal fluctuations impact metabolite sensing [188,189]. Future translational research must prioritize the use of Humanized-Microbiome mouse models engrafted with female patient flora and subject to cycling hormone replacement, or utilize vagina-on-a-chip organoid systems that integrate epithelial, immune, and microbial components to test metabolic interventions under controlled hormonal condition [180,190,191].

### 6.2. Defining Clinical Metabotypes: A New Diagnostic Taxonomy

To bridge the translational gap between mechanistic research and clinical implementation, we categorize female immune dysregulation into three distinct metabotypes. These categories, detailed in Table 1, represent specific failures in the host-microbiota metabolic integration and require stratified therapeutic modalities tailored to the underlying genetic and microbial landscape.

Metabotype A: The SCFA-Deficient/High-Xist Responder

Definition: This phenotype characterizes a subset of female autoimmune patients (e.g., SLE, RA) who exhibit a double hit: (1) High titers of autoantibodies against Xist-RNP complexes (indicating strong genetic intrinsic priming), combined with (2) A compromised butyrate synthesis capacity, reflected by either low fecal concentrations (<10 mmol/kg) or a metagenomic depletion of the key synthesis gene, butyryl-CoA:acetate CoA-transferase [186,192,193].

Clinical Significance: In these patients, the epigenetic brake (HDAC inhibition) is structurally absent [155]. Consequently, the Xist-driven innate activation proceeds unchecked.

Precision Intervention: Standard immunosuppressants (e.g., hydroxychloroquine) may be insufficient. These patients are ideal candidates for High-Dose Postbiotic Therapy (direct delivery of colonic-release butyrate salts) or Resistant Starch Supplementation (e.g., HAM-RS2) to selectively bloom *Faecalibacterium prausnitzii* [194]. Recent reviews emphasize that diet-microbiota interactions are highly individualized; Thus, confirming the expansion of butyrate producers post-intervention is mandatory [195,196]. Crucially, efficacy should be monitored not just by symptom relief, but by the restoration of H3K27ac levels in circulating regulatory T cells [197].

Metabotype B: The Non-Equol Producer

Definition: This metabolic trait divides the female population into Producers (approx. 30–50%) and Non-Producers, defined by the presence of specific bacterial genera like *Slackia* and Adlercreutzia capable of converting dietary daidzein into S-Equol [198].

Clinical Significance: As detailed in Section 4.3, S-Equol is a potent, selective ERβ agonist that reinforces gut barrier integrity and limits inflammation. Non-Producers are likely unresponsive to the benefits of dietary soy. In a clinical trial setting, pooling these two groups leads to statistical null results, masking the efficacy in producers [199].

Precision Intervention: For Non-Producers, dietary advice to consume more soy is clinically ineffective. Instead, this metabotype necessitates Direct Ligand Replacement through the supplementation of pharmacologically stabilized S-Equol, thereby bypassing the requirement for microbial transformation. Alternatively, Next-Generation Probiotics (NGPs) consisting of engineered *Slackia* strains could be administered to permanently colonize the gut and restore metabolic competence [200,201].

Metabotype C: The Lactate-Depleted/Succinate-High

Definition: This phenotype is specific to the vaginal mucosa, characterized by the loss of *Lactobacillus crispatus* and the accumulation of succinate-producing anaerobes (*Prevotella*, *Mobiluncus*), often misdiagnosed as recurrent yeast infections or asymptomatic dysbiosis [201,202].

Clinical Significance: The loss of lactate removes the lactate lock on local dendritic cells and prevents Histone Lactylation (H3K18la) in mucosal Treg [84]. The concurrent rise in succinate stabilizes HIF-1α, driving inflammation. This state is a primary risk factor for preterm birth and HIV acquisition [190,203].

Precision Intervention: Antibiotics (metronidazole) often fail to restore the *Lactobacillus* dominance due to biofilm formation. The emerging standard for this metabotype is Vaginal Microbiota Transplantation (VMT) from a *L. crispatus*-dominant donor, or the use of L-lactate-containing acidifying gels specifically timed to the post-menses window to re-seed the metabolic shield [204,205].

### 6.3. The Precision Window: Cycle-Synced Immunotherapy

Perhaps the most critical, yet ignored, aspect of female immunity is the menstrual cycle [206]. The host’s ability to sense and utilize microbial metabolites fluctuates with hormonal phases [207]. We propose a Cycle-Synced intervention strategy.

The Follicular Phase (High Estrogen/Low Progesterone):

Physiological Context: High estradiol (E2) can downregulate the expression of the butyrate transporter SMCT1 (Slc5a8) in the gut epithelium, creating a transient resistance to microbial signals [117]. Simultaneously, E2 promotes Th1/Th17 dominance [177,208].

Therapeutic Window: This is the window of vulnerability. During the late follicular phase (ovulation), female patients with autoimmune diathesis may require significantly higher doses of SCFAs or AhR ligands to overcome transporter downregulation and dampen the estrogen-driven inflammatory spike. Specifically, microbial AhR ligands have been shown to suppress arthritis by amplifying AhR activation in regulatory B cells, a mechanism that may need boosting during estrogen peaks [209].

The Luteal Phase (High Progesterone):

Physiological Context: Progesterone naturally upregulates mucosal barrier function and synergizes with Tregs.

Therapeutic Window: This is the window of opportunity for colonization. The tolerogenic environment may be more permissive to the engraftment of live biotherapeutics (e.g., Faecalibacterium or *Lactobacillus*). Interventions aimed at restoring the ecosystem (probiotics/FMT) may have higher success rates if performed during the mid-luteal phase rather than the inflammatory follicular phase [208,210,211].

### 6.4. Technical Roadblocks: From Relative to Absolute Quantification

Implementing this metabotyping requires a paradigm shift in diagnostics. Current 16S rRNA sequencing provides only relative abundance (percentages), which correlates poorly with the actual metabolic output (micromolar concentrations) of the microbiota [212,213]. A patient might have “20% *Lactobacillus*,” but if the total bacterial load is low, the absolute lactate concentration may still be insufficient to drive histone lactylation (which requires > 5 mM intracellularly). To illustrate this translational critical gap, consider a theoretical clinical scenario: a patient’s mucosal swab might reveal a microbiome comprising 20% *Lactobacillus*. However, if the absolute bacterial load is severely depleted (e.g., post-antibiotic therapy), the localized mucosal lactate concentration may fail to reach the millimolar thresholds—such as the ~5 mM physiological range often required to drive robust p300-dependent histone lactylation in vitro [214,215,216]. Thus, relative abundance readouts obscure the true metabolic competence required to induce epigenetic rewiring in host immune cells.

Future clinical protocols must integrate quantitative metabolomics to measure absolute concentrations of butyrate, S-Equol, and L-lactate in serum or stool alongside metagenomics [217]. Furthermore, resolving specific isomers is critical; for instance, distinguishing between L-lactate (beneficial, host-compatible) and D-lactate (potentially toxic, produced by pathobionts) is essential to avoid misinterpretation of high lactate states [84,218]. The development of Smart ingestible sensors capable of real-time monitoring of gut gas profiles and metabolite levels offers a promising avenue for continuous, isomer-specific metabotyping [219]

### 6.5. Limitations and Alternative Explanations

While the interaction between Xist RNPs and microbial metabolites offers a compelling framework, it is important to acknowledge the limitations of current evidence. First, the causality between dysbiosis and autoimmunity remains bidirectional; it is plausible that systemic inflammation drives the depletion of butyrate producers rather than vice versa [220]. Second, alternative mechanisms, such as the direct modulation of immune genes by sex hormones independent of metabolic intermediates, play a significant role that cannot be subsumed under this hypothesis [221]. Finally, not all female-biased autoimmune diseases fit this model perfectly; for instance, in Multiple Sclerosis, the role of specific gut metabolites remains more controversial compared to SLE [222,223]. Future research must rigorously disentangle these overlapping drivers.

### 6.6. Conclusion of the Framework

In conclusion, the path to managing female immune pathologies lies in recognizing that the female immune system is a Superorganism operating under distinct evolutionary constraints. Transitioning from generic probiotic approaches to a sophisticated strategy of Sexual Dimorphic Metabotyping offers a path to restore the metabolic calibration intended by evolution. This precision approach relies on diagnosing specific metabolic deficits, for instance in non-equol producers, and timing interventions to the menstrual cycle. Recent perspectives underscore that ignoring these sexual dimorphisms in the gut microbiome leads to fundamentally flawed precision medicine approaches. This represents not just a new treatment modality, but a fundamental reimaging of women’s health as a dynamic integration of genome, hormone, and microbiome.

## 7. Conclusions

The Metabolic Calibration Hypothesis posits that female immune homeostasis is sustained by a co-evolved dependency between X-linked genetic drivers and microbiota-derived regulatory signals. This integrated architecture facilitates the immunological plasticity required for physiological tolerance during pregnancy, yet it predisposes the female host to autoimmune dysregulation when microbial metabolic outputs, particularly butyrate and L-lactate, fail to stabilize intrinsic Xist-mediated priming. Current microbiome-based interventions frequently overlook these sex-specific inflammatory pressures and the unique metabolic constraints imposed by estrogenic signaling. Consequently, advancing women’s immune health necessitates a transition toward Sexual Dimorphic Metabotyping, a precision framework designed to stratify patients according to their metabolic competence and holobiont potential. By defining the microbiota as an essential epigenetic regulator of the X chromosome, therapeutic strategies can progress from generic supplementation toward the precise restoration of physiological tolerance, thereby addressing the distinct evolutionary architecture of the female immune system.

## Figures and Tables

**Table 1:** Clinical Metabotypes and Precision Intervention Strategies.

Clinical Metabotype	Key Diagnostic Features	Precision Intervention Strategy
Metabotype A: SCFA-Deficient and Xist-Sensitized	Elevated anti-Xist RNP autoantibody titers; fecal butyrate concentration below 10 mmol/kg; metagenomic depletion of the but gene; diminished H3K27ac enrichment at the Foxp3-CNS1 locus in circulating Tregs.	-Intervention: Administration of high-dose colonic-release butyrate salts or HAM-RS2 starch supplementation. -Therapeutic Monitoring: ChIP-qPCR quantification of H3K27ac enrichment; assessment of circulating Treg frequency and suppressive capacity in ex vivo co-culture assays.
Metabotype B: Equol Non-Producing Phenotype	Genomic absence of Slackia and Adlercreutzia species; plasma S-Equol concentrations below 10 ng/mL following a standardized daidzein challenge; history of suboptimal response to soy-based dietary interventions.	-Intervention: Direct supplementation with pharmacologically stabilized S-Equol; utilization of next-generation probiotics featuring engineered equol-producing strains. -Therapeutic Monitoring: Achievement of plasma S-Equol concentrations between 50 and 100 ng/mL via LC-MS/MS; reduction in serum markers of gut permeability.
Metabotype C: Lactate-Depleted and Succinate-High	Anaerobe-dominant vaginal microbiome; L-lactate levels below 20 mM alongside succinate accumulation via targeted metabolomics; depletion of *Lactobacillus crispatus*; diminished H3K18la marks on cervical epithelial cells.	-Intervention: Vaginal Microbiota Transplantation (VMT) from an *L. crispatus* dominant donor; application of L-lactate acidifying gels specifically during the post-menses window. -Therapeutic Monitoring: Quantification of H3K18la levels via LC-MS/MS histone analysis; restoration of an *L. crispatus* dominant microbiome with a Nugent score of 3 or less.

## Data Availability

No new data were created or analyzed in this study. Data sharing is not applicable to this article.

## Abbreviations

ACR	American College of Rheumatology
AF-1	Activation Function 1
AUC	Area Under the Curve
BMDM	Bone Marrow-Derived Macrophage
BTK	Bruton’s Tyrosine Kinase
CA19-9	Cancer Antigen 19-9
CCDS	Consensus Coding Sequence
CD40L	CD40 Ligand
CEA	Carcinoembryonic Antigen
CXCR3	C-X-C Motif Chemokine Receptor 3
CXorf21	Chromosome X Open Reading Frame 21
CYBB	Cytochrome b-245 Beta Chain
CZB	Chatot, Ziomek, and Bavister
DCA	Dichloroacetate
FOXP3/FoxP3	Forkhead Box P3
FMT	Fecal Microbiota Transplantation
GnT1IP-L	N-acetylglucosaminyltransferase I Inhibitory Protein Large Form
HAM-RS2	Histone Acetylation/Methylation Reference Standard 2
H2AK119-Ub	Histone H2A Lysine 119 Ubiquitination
H3K18	Histone H3 Lysine-18
H3K18ac	Histone H3 Lysine-18 Acetylation
H3K18la	Histone H3 Lysine-18 Lactylation
H3K27ac	Histone H3 Lysine-27 Acetylation
H3K27me3	Histone H3 Lysine-27 Trimethylation
H3K4me3	Histone H3 Lysine-4 Trimethylation
HOLI	Hormone Receptor Ligand-Binding Domain
hPTMs	Histone Post-Translational Modifications
IFN-I	Type I Interferon
IFNγ	Interferon Gamma
IL2RA	Interleukin 2 Receptor Subunit Alpha
Il2rg	Interleukin 2 Receptor Subunit Gamma
IL7RA	Interleukin 7 Receptor Subunit Alpha
IRAK1	Interleukin-1 Receptor-Associated Kinase 1
KDM6A/UTX	Lysine Demethylase 6A/Ubiquitously Transcribed Tetratricopeptide Repeat, X Chromosome
KSOM	Potassium Simplex Optimized Medium
LDHA	Lactate Dehydrogenase A
LDHB	Lactate Dehydrogenase B
LPS	Lipopolysaccharide
MGAT1	Mannosyl (alpha-1,3-)-glycoprotein beta-1,2-N-acetylglucosaminyltransferase
MS	Multiple Sclerosis
MZT	Maternal-to-Zygotic Transition
NEMO	NF-kappa-B Essential Modulator
NGP	Neutrophil Granulocyte Precursor
NR4A1	Nuclear Receptor Subfamily 4 Group A Member 1
Pan-Kla/Kla	Pan Lysine Lactylation
pDC	Plasmacytoid Dendritic Cell
QoL	Quality of Life
SELENA	Safety of Estrogens in Lupus Erythematosus National Assessment
SLC35A2	Solute Carrier Family 35 Member A2
SLC35A3	Solute Carrier Family 35 Member A3
SLE	Systemic Lupus Erythematosus
SLEDAI	Systemic Lupus Erythematosus Disease Activity Index
SLICC	Systemic Lupus International Collaborating Clinics
SRI	SLE Responder Index
T1D	Type 1 Diabetes
TLR7	Toll-Like Receptor 7
TLR8	Toll-Like Receptor 8
VMT	Vaginal Microbiota Transplantation
Xa	Active X Chromosome
XCI	X Chromosome Inactivation
Xi	Inactive X Chromosome
Xist/XIST	X-Inactive Specific Transcript
ZGA	Zygotic Gene Activation
ZnF_C4	Nuclear Hormone Receptor Zinc Finger Domain
